# Concentration shift experiment with an electrode of active material for precise electrochemical analysis†

**DOI:** 10.1039/d3ra03630h

**Published:** 2023-07-19

**Authors:** Tamotsu Sawahashi, Koji Hiraoka, Shiro Seki

**Affiliations:** a Graduate School of Applied Chemistry and Chemical Engineering, Kogakuin University 2665-1 Nakano-machi Hachioji Tokyo 192-0015 Japan shiro-seki@cc.kogakuin.ac.jp +81-42-628-4568 +81-42-628-4568

## Abstract

To precisely evaluate the electrochemical properties of a battery of active material, we proposed a “concentration shift experiment” using single-particle electrochemical measurement (SPEM) and a diluted electrode sheet (DES). SPEM can be used for information, such as the charge–discharge and resistance properties of only the active material (extremely dilute condition: ≈0). DES consists of concentrations varying from 1% to 100% of the active material (LiCoO_2_) and inactive material (α-Al_2_O_3_), electrically conductive additive and binder polymer onto an Al current collector. The resistance components derived from the LiCoO_2_ single particles were measured and calculated. Their apparent activation energy (*E*_a_) was 27 kJ mol^−1^, which is relatively low compared with the applied-type sheet electrode (30–60 kJ mol^−1^). Simple electric/ionic conductive route was analyzed using SPEM cell, and the fundamental LiCoO_2_ originated *E*_a_ could be calculated. Resistance components attributed to LiCoO_2_ were also measured and extracted by alternating current impedance measurements using DES. The resistance non-linearly decreased with LiCoO_2_ concentration, and the percolation and inhomogeneity of LiCoO_2_ particles were suspected. The planful isolation of an active material particle should be critical for the overall information on an electrode particle.

## Abstract 2

To precisely evaluate the electrochemical properties of a battery of active material, we proposed a “concentration shift experiment” using single-particle electrochemical measurement (SPEM) and a diluted electrode sheet (DES).

## Graphical abstract

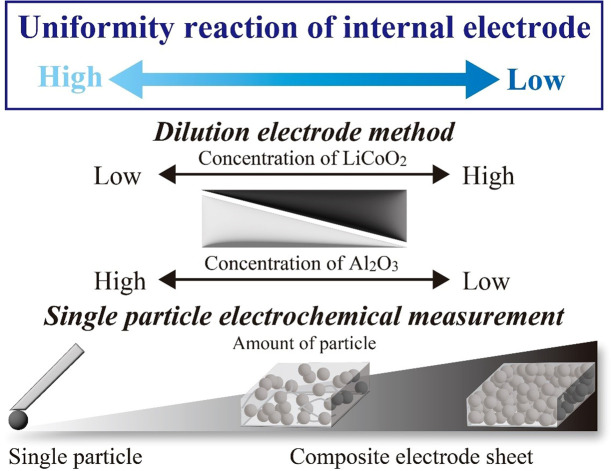

## 1. Introduction

Recently, Li-ion batteries (LIBs) have reached a practically usable stage for consumer use (laptop PC and smartphone), transportation (electric vehicle),^1,2^ and large-scale energy storage devices for unstable renewable energy and are expected to be a key technology in the realization of a low-carbon society.^3–6^ In general, energy densities, such as electrical capacity, operating voltage, and input–output properties, are among the most critical parameters for the improvement of LIBs to expand the usage of battery systems. Therefore, various approaches for battery improvement have been explored by analyzing each component (positive and negative electrodes and electrolytes) and their combinations and optimizations.^7–9^ In particular, electrode active material particles always act as charge–discharge media, which converts between chemical and electrical energies with electrolyte-mediated conditions in electrochemical cells. However, electrode materials used in conventional LIBs are usually prepared as composite materials and also consist of active materials, electrically conductive additives based on carbon materials, and binders based on good bonding/forming polymer materials. Composite electrode materials pose various difficulties, not only for the precise electrochemical observation of active materials but also for the correct evaluations of capacity and resistance degradations for LIBs by the inhomogeneous reaction distributions of thickness/in-plane directions with charge–discharge operations.^10–12^ Therefore, electrochemical analysis methods for evaluating the charge–discharge behaviour and reactive uniformity of active materials will be critical apart from the electrochemical properties of functional materials to extract the factors governing battery performance.

**Fig. 1:**
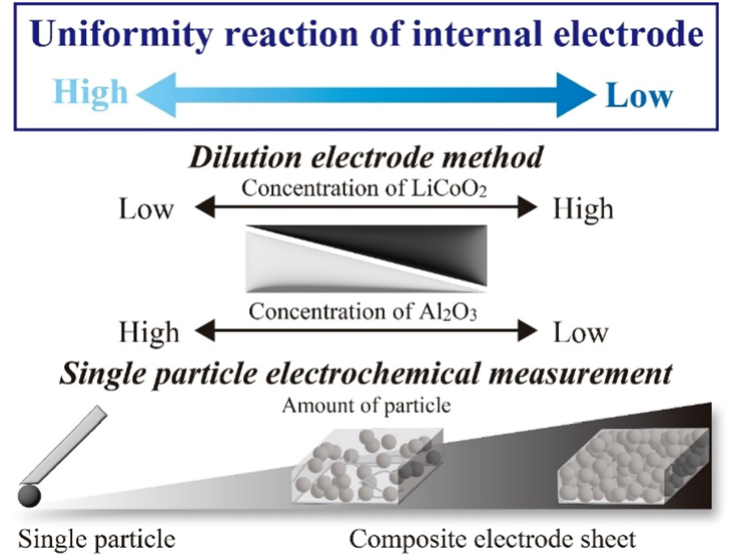
Image of the analysis of active material using dilute electrode sheet and single-particle electrochemical measurement.

To obtain quantitative electrochemical information on the electrode of active materials, single-particle (SP) electrode measurements (SPEMs) method have been proposed using positive active material LiCoO_2_ (LCO).^13–17^ A microprobe is directly attached to the SP and electrochemically operated for SPEM measurements, and charge–discharge behaviour and resistance properties are originated by an electrode particle as the “smallest” cell system without other components. Dokko *et al.* reported the electrochemical properties of LCO-SP during the intercalation/deintercalation processes of Li ions in LCO.^13^ We also recorded a ‘‘concentration shift experiment’’ by comparing the resistance components between SPEM cells and coin cells (Fig. 1), which enabled the reasonable estimation of particle counts in electrode sheets.^15^ In addition, Ariyoshi *et al.* reported the diluted electrode sheet (DES) method using inactive inorganic particles having similar morphologies as “quasi” components in electrode sheets and clarified the rate properties of Li[Li_0.1_Al_0.1_Mn_1.8_]O_4_.^18^ In this study, the reaction processes (including reaction and transport routes) of LCO were investigated from the viewpoint of concentration shift of active materials using both SPEM (infinitely dilute condition of active materials) and DES methods by varying the LCO/α-Al_2_O_3_ contents and their resistance components attributed to LCO were evaluated using alternating current (AC) impedance measurements.^19–21^ Therefore, we investigated the consistency and uniformity of the SPEM and DES methods in terms of resistance and frequency (time constant) components.

## 2. Experimental

### 2.1 Preparation of microelectrodes and measurement of SPEM cells

**Fig. 2:**
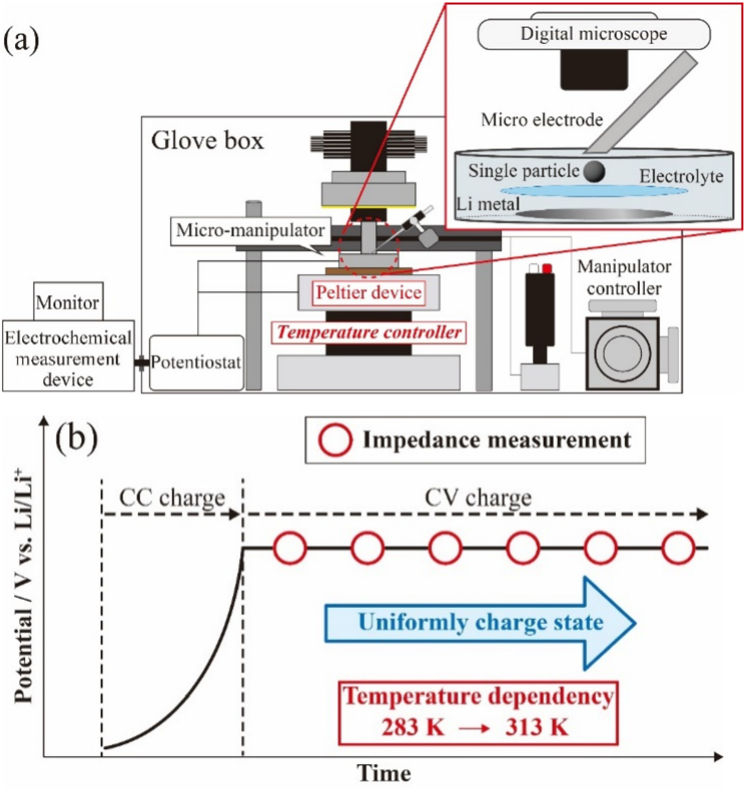
Equipment setup of single particle electrochemical measurement (a), experimental flow of AC impedance measurement (b).

To prepare the microelectrode for the measurements,^13^ a glass capillary (G-100L, inside diameter: 0.75 μm, Narishige) was heated and pulled using a puller (Narishige, PC-10) and obtained acuate apex. Thin Pt wire (*ϕ* 30 μm, Nilaco) and thick Cu wire (*ϕ* 0.1 mm, Nilaco) were welded using a spot welding machine (KTH-MWS, Kondo Tech) to obtain self-standing metal wires. The hybridized wire was inserted into the glass capillary to the melted apex and was fused using a microforge (MF-900, Narishige). For accurate electrical contact with the active material particle, the microelectrode tip was polished at 45° using a rotary polishing machine (EG-402, Narishige). Fig. 2(a) shows a schematic image of the SPEM system used in this study, and electrochemical measurements were carried out in an argon-filled glovebox (DBO-1B, Miwa Manufacturing, [O_2_] < 10 ppm, dew point < 193 K). A Li foil disk (*ϕ* 12 mm, Honjo Metal) was attached to a stainless steel cup as the negative (counter and reference) electrode. LiCoO_2_ particles (Honjo Chemical) were dispersed onto a glass filter (GC-50, Advantec) as the positive (working) electrode. The electrolyte, comprising 1.0 mol kg^−1^ LiN(SO_2_F)_2_ (LiFSA, Nippon Shokubai)/ethylene carbonate (EC) electrolyte solution, was dropped onto this two-electrode cell for SPEM. After that, the prepared microelectrode and an LCO particle were attached. Electrical conduction confirmed under observation with a digital microscope, and various electrochemical measurements were conducted.

### 2.2 Preparation of dilute-type applied electrode sheet and coin cell

To investigate the concentration effect for active materials of the electrode sheet, an applied-type electrode sheet containing various concentrations of LCO as active material and α-Al_2_O_3_ as a blank material was used. A slurry in which LCO and α-Al_2_O_3_ particles (Koujundo Chemical), acetylene black (Li-100, AB, Denka), and polyvinylidene fluoride (PVdF, Kureha) were mixed at a mass ratio of 84 : 10 : 6 = (LCO + α-Al_2_O_3_) : AB : PVdF was applied to an Al foil current collector. In addition, the weight ratio (wt%) of LCO toward (LCO + α-Al_2_O_3_) was varied using 1.0, 5.0, 10, 20, 40, 60, 80, and 100 wt% by using a paste mixer (UM-113S, Japan Unix). Then, *N*-methyl-2-pyrrolidone was added to ensure a homogeneous slurry and reduce the mixture viscosity. The prepared electrode sheets were dried for 12 h in a thermostatic oven at 333 K under vacuum conditions, pressed and punched into *ϕ* 16 mm diameter disks (average porosity: *ca.* 60%, SEM images: Fig. S1†). [Li|electrolyte|LCO electrode sheet] cells were prepared by encapsulating the prepared electrode sheet, a glass separator (GA-55, Advantec), 1.0 mol kg^−1^ LiFSA-EC electrolyte solution, and Li metal in a 2032-type coin cell in an argon-filled glovebox.

### 2.3 Electrochemical evaluations for SPEM and coin cells

To evaluate the electrochemical properties of LCO-SPs, charge–discharge and AC impedance measurements were examined following the scheme as shown in Fig. 2(b). An SP of LCO (diameter: *ca* 20–30 μm) was charged to 3.95 V *vs.* Li/Li^+^ under 3 nA constant-current and then charged at a constant-voltage for more than 1200 s (current decayed less than 300 pA) at ambient temperature. AC impedance measurements were performed in the frequency range of 20 kHz to 10 mHz at 3.95 V *vs.* Li/Li^+^ under an applied voltage of 50 mV at temperatures of 283.9, 286.8, 293.0, 297.5, 302.9, 308.0, and 313.3 K using a specially attached Peltier unit (Fig. S2†). By contrast, the prepared coin cells were also charged to 3.95 V *vs.* Li/Li^+^ under 20 μA (10 μA cm^−2^) constant-current constant-voltage condition, and AC impedance measurements were performed in the frequency range of 500 kHz to 10 mHz at 3.95 V *vs.* Li/Li^+^ under an applied voltage of 200 mV at 303.2 K.

## 3. Results and discussion

### 3.1 Electrochemical measurements for LiCoO_2_ single particles

**Fig. 3:**
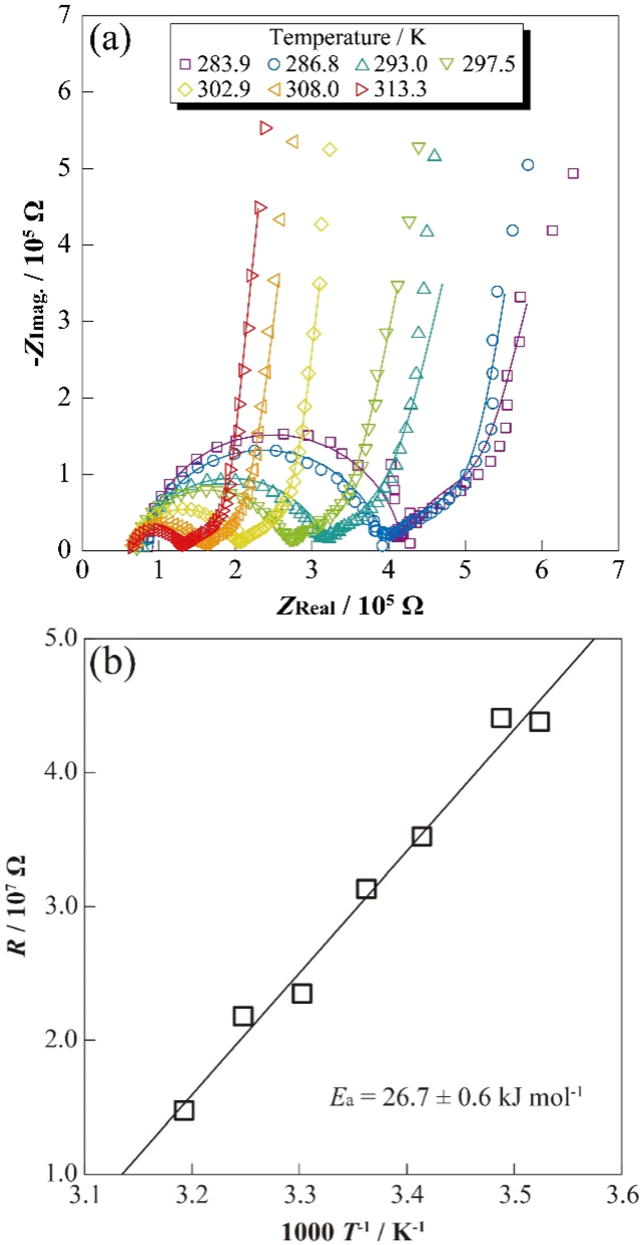
Nyquist plots for a single-particle LiCoO_2_ in a SPEM cell at 286.8–313.3 K (a), Arrhenius plots of resistance for a LiCoO_2_ single particle (b) at 3.95 V *vs.* Li/Li^+^.

To evaluate the correlation between temperature and the resistance properties of charged LCO-SPs (Li_*x*_CoO_2_: *x* < 1.0), the temperature dependence of AC impedance spectra of SP electrode cells was measured. The temperature dependence of impedance spectra for [Li|electrolyte|LCO-SP] cell at 3.95 V *vs.* Li/Li^+^ are shown in Fig. 3(a). A semicircular arc (500 Hz to 1.26 kHz) was observed for each spectrum, which suggested several resistance components derived from the bulk of LCO-SP and Li^+^ transfer at the electrode/electrolyte interface^13^ owing to their slight asymmetry properties for the overlapped time constant of each reaction/transfer process. The decrease in diameter for the obtained semicircular arcs was confirmed with temperature increase, as in the case of applied-type electrode sheets^22,23^ attributed to the acceleration of Li^+^ transfers and decrease in resistance. Moreover, the resistance component suggesting a Warburg component inclining 45° was confirmed, and their temperature dependences were also smaller than for the low-frequency semicircular arc. Therefore, the inclining Warburg component might be considered, for example, solid-electrolyte-interphase film resistance by the oxidative decomposition of the electrolyte solution with LCO charging. In this case, the resistance component derived from Li metal interfaces should be negligible because of their differences in the physical reaction area of electrodes (Li metal: *ca.* 2 cm^2^). In addition, the response current should passage into/surface LCO-SP, which differs from the case of applied sheets containing, for example, electrically conductive additive and binder polymer (several conducting routes). Thus, the obtained temperature dependences of impedance spectra were assumed reflecting the electrochemical responses of only LCO-SP.

To separate the resistance components of impedance spectra for the [Li|electrolyte|LCO-SP] cell, the equivalent circuit described by eqn (1) was assumed, and a fitting analysis was performed.

1 *L*_S1_/*R*_S1_ + *R*_S2_ + *Q*_S3_/*R*_S3_ + *Q*_S4_/*R*_S4_ + *Q*_S5_/*R*_S5_,

where *L*_S1_ and *R*_S1_ are the inductance and resistance components attributed to the measurement environment, *R*_S2_ is electrolyte bulk resistance, *R*_S3_, *R*_S4_, and *R*_S5_ are the resistance components attributed to the LCO-SP (including bulk, interface, and diffusion components), and *Q*_S3_, *Q*_S4_, and *Q*_S5_ are the quasi-capacitance components of each resistance. Among the obtained impedance spectra, *R*_S3_ and *R*_S4_ were suggested as the internal resistance of the LCO particle and interfacial resistance at the LCO/electrolyte (both with information for LCO), respectively, from the frequency responses. However, it is difficult to determine the precise assignment for each resistance component, and therefore, the sum of *R*_S3_ and *R*_S4_ was defined as the resistance concerning the transport and reaction process of LCO-SP (*R*_LCO-SP_). Fig. 3(b) shows the Arrhenius-type temperature dependences for *R*_LCO-SP_ of the [Li|electrolyte|LCO-SP] cell. Highly linear relationships between the inverse of temperature and *R*_LCO-SP_ were obtained, and also the apparent activation energy (*E*_a_) was calculated using the Arrhenius equation as follows:

2 *k* = *A* exp(−*E*_a_/*RT*),

where *k*, *A*, *E*_a_, *R*, and *T* are the reaction rate constant, frequency factor, activation energy, gas constant, and absolute temperature, respectively. The apparent *E*_a_ calculated from eqn (2) was 26.7 ± 0.6 kJ mol^−1^ with a relatively low activation barrier. The *E*_a_ values of interfacial resistances of applied-type electrodes using active material (such as LiCoO_2_, LiFePO_4_, and LiMn_2_O_4_), electrically conductive additive, and binder polymer for conventional Li-ion cells have been reported to be between 30 and 60 kJ mol^−1^,^22,24–26^ and also included several electrochemical processes, such as internal active material and charge transfer at each component. The complication for Li^+^ transport properties due to the introduction of various materials into the electrode sheet should result in increasing trends of *E*_a_ compared with the case of SP, and a lower *E*_a_ value was obtained using a simple route with only LCO and electrolyte solution.

### 3.2 Evaluation of the concentration effect for LiCoO_2_ electrode sheet

**Fig. 4:**
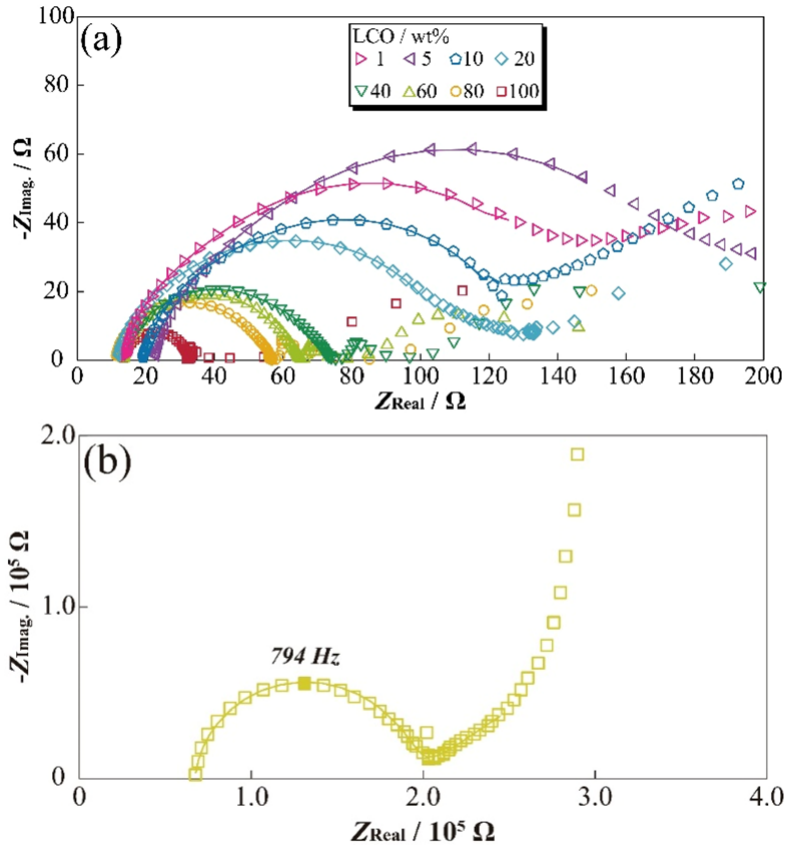
Nyquist plots for an [LiCoO_2_-DES|1 mol kg^−1^ LiFSA/EC|Li metal] coin cell for changing the wt% ratio of active material (a), and a single-particle LiCoO_2_ in a SPEM cell (b) at 308.0 K at 3.95 V *vs.* Li/Li^+^.

To evaluate the concentration effect on the applied electrode sheet, DESs^18,27^ were prepared by mixing α-Al_2_O_3_ powder as the inactive materials with varying LCO concentrations. The prepared [Li metal|electrolyte|LCO-DES] cells were charged to 3.95 V *vs.* Li/Li^+^, and then AC impedance measurements were attempted at 3.95 V *vs.* Li/Li^+^. A stable electrode plateau was obtained around 3.92 V *vs.* Li/Li^+^, which attributed to the charging process (deintercalation) of LCO, even though the charge capacity of DES monotonically decreased with the Al_2_O_3_ amount (Fig. S3†). Fig. 4(a) shows the LCO concentration dependences for the impedance spectra of [Li metal|electrolyte|LCO-DES] cells at 303.2 K. A semicircular arc was observed from each impedance spectrum (*ca.* 500 Hz–1.26 kHz) and was also shifted to asymmetric shape and high resistance in the low-frequency region with an increase in the α-Al_2_O_3_ amount. From the previous studies,^28,29^ the impedance spectra of LCO positive electrode and Li metal negative electrode can be separated at low- and high-frequency using the differences of each time constant for the reactions. Therefore, changes in the semicircular arc at low-frequency might suggest a decrease in absolute reaction area for LCO by the introduction of α-Al_2_O_3_ as a fake electrode particle. Fig. 4(b) also shows the impedance spectra of a [Li|electrolyte|LCO-SP] cell at 303.2 K, which exhibited a highly symmetric semicircular arc about 1000 times larger than for [Li metal|electrolyte|LCO-DES] cells owing to their differences in the absolute amount of LCO.

**Fig. 5:**
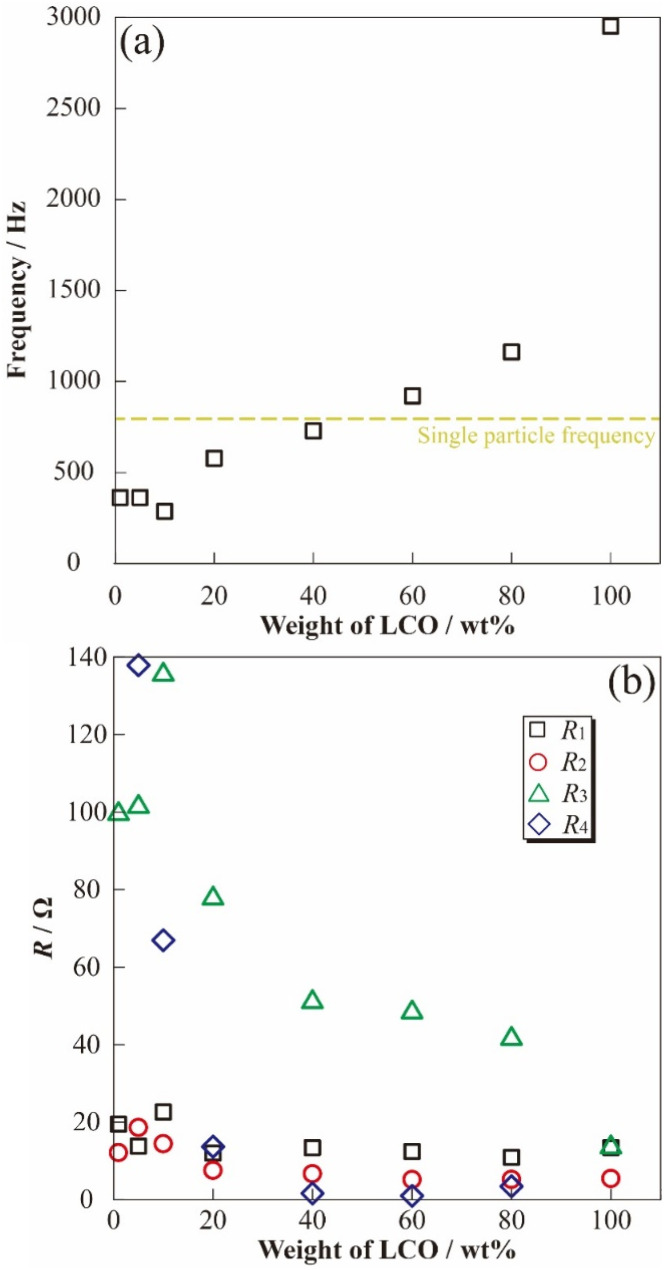
Correlation of active material ratio and frequency dependences (a), each resistance component (b) at 3.95 V *vs.* Li/Li^+^.

To clarify the effect of LCO concentration on the electrode sheet for the time constant of electrode reactions, relationships between the LCO content of DES and apex frequency related to the time constant are shown in Fig. 5(a). The apex frequency decreased with decreasing LCO concentration in [Li metal|electrolyte|LCO-DES] cells with minimum local region (1–10 wt%, percolated concentration of LCO). Therefore, the change in LCO concentration affected not only the interfacial resistance related to the reaction area but also the reaction speed (processes) of the electrode reactions owing to their inclusion of electric, conductive, additive, and binder polymer (length of reaction route). In addition, in the case of less than 40 wt% of LCO, the [Li metal|electrolyte|LCO-DES] cells exhibited higher apex frequency than the [Li|electrolyte|LCO-SP] cell (yellow dashed line as shown at *x* ≈ 0). Changes in frequency properties should be correlated with the reaction processes of LCO (contribution of Li metal might not be included) and approach to the phenomena of the SP by decreasing the LCO amount and concentrations. In particular, slight apex frequency (less than 40 wt% of LCO) suggested a change in electron/lithium conducting path and interface at the electrode/electrolyte boundary, including another component such as electrically conductive additive and binder polymer. To analyze changes in the resistance components from the observed semicircular arc of the impedance spectra, fitting analyses were performed using the following eqn (3) to obtain well-fitted parameters for analysis,

3 *R*_1_ + *Q*_2_/*R*_2_ + *Q*_3_/*R*_3_ + *Q*_4_/*R*_4_,

where, *R*_1_, *R*_2_, *R*_3_, and *R*_4_ are the bulk resistance of electrolyte solution, resistance components attributed to the negative electrode, positive electrode (including α-Al_2_O_3_ contribution), and the related *Q* is their quasi-capacitance components, respectively. Each calculated *R* value is depicted in Fig. 5(b) as the dependence of the weight percentage of LCO. Although *R*_1_ and *R*_2_ exhibited almost constant values (nearly less than 20 Ω), positive electrode-related *R*_3_ and *R*_4_ nonlinearly decreased with LCO concentration. In particular, *R*_4_ rapidly increased with the amount of α-Al_2_O_3_, and were considered to correlate with the continuous/percolation route of Li^+^ into positive electrode sheet. The result showed that both the electrolyte and negative electrode resistances are not dependent of the α-Al_2_O_3_ introduction into positive electrode sheet materials. Conversely, *R*_4_ was not exhibited and included the composition of LCO: 100 wt%, and the resistance component should be assigned as the parameters derived from the change of conducting route into the positive electrode sheet and their uniformity (absence of α-Al_2_O_3_). To evaluate the effects of α-Al_2_O_3_ introduction into the LCO electrode sheet from the viewpoint of morphologies, scanning electron microscope images of each material (powder and sheet) were observed (Fig. S2†). Differences in the electrically conductive properties gave the light and dark contrast, composed of LCO and α-Al_2_O_3_ assigned as light and dark particles, respectively, and aggregates of LCO were also confirmed in the LCO: 40 wt% sample. Electrode morphologies can be changed by introducing α-Al_2_O_3_ particles owing to their composition and continuous phase changes by an excess amount of α-Al_2_O_3_. In other words, the existence of inactive α-Al_2_O_3_ (*i.e.*, more than 60 wt%) should also affect the *R*_4_ owing to their changes in the percolation conductive model for LCO. In addition, the *R*_3_ also decreased with LCO concentrations and was also considered to correlate with the number of LCO particles (apparent reactive area of LCO). In particular, in the dilute region of LCO particles (less than 40 wt% of LCO), the electrochemical information of electrode particles might be determined, owing to their independence of electrochemical active part without aggregations similar to the case of the SP measurements. Inactive α-Al_2_O_3_ acted with sufficiently dilute media and blank materials to basically evaluate systems for precise resistance components of the LIBs. In the future, we will investigate the correlation of electrochemical and spectroscopic properties (*e.g.*, Raman spectrum) to evaluate the precise reaction mechanism of electrodes for battery materials.

## 4. Conclusions

The precise reactive resistance of LCO was evaluated by AC impedance measurement using SPEM and DES cells with the varying ratios of LCO and α-Al_2_O_3_. The results of this study are summarized as follows:

(1) Arrhenius-type plots of resistance component derived from LCO-SP (*R*_LCO-SP_) exhibited clear linear trends against inverse temperature, and the calculated *E*_a_ was 26.7 ± 0.6 kJ mol^−1^. A relatively low *E*_a_ value was obtained by the simple electric/ionic conductive route differing from the conventional electrode sheets, which consists of other materials.

(2) Resistance components attributed to positive active materials, were well separated into two components (*R*_3_ and *R*_4_) in [Li metal|electrolyte|LCO-DES] cells. The existence of *R*_4_ was confirmed at less than 20 wt% of LCO, although *R*_4_ was also not observed at 100 wt%. Therefore, *R*_4_ was suggested as the parameter for the electric/ionic route (continuous phase) and its uniformity in the electrode sheet. By contrast, *R*_3_ decreased with LCO concentrations and was firmly dependent on reactive area and the number of LCO particles.

As mentioned earlier, a correlation investigation of SPEM and DES measurements enabled a unified electrochemical analysis of electrode active materials for battery systems. In the future, we will report the SPEM and DES results using another active material for the negative electrode (Li_4_Ti_5_O_12_ and C_6_), chemically additive, and spectroscopic analysis by Raman spectroscopy to determine the active/inactive part of electrode materials, such as both the SP and electrode sheet.

## Conflicts of interest

There are no conflicts of interest to declare.

## Supplementary Material

RA-013-D3RA03630H-s001
