# Supplementary material for: Concentration shift experiment with an electrode of active material for precise electrochemical analysis
Source: RSC Adv. 2023 Jul 19;13(31):21667–72. doi: 10.1039/d3ra03630h (PMC10354599; doi:10.1039/d3ra03630h)
Supplement: RA-013-D3RA03630H-s001 [file RA-013-D3RA03630H-s001.pdf]

Electronic Supplementary Material (ESI) for RSC Advances.
This journal is © The Royal Society of Chemistry 2023

Electronic Supplementary Information (ESI)

Concentration shift experiment with electrode active material for precise electrochemical analysis

Tamotsu Sawahashi, Koji Hiraoka, Shiro Seki*

Graduate School of Applied Chemistry and Chemical Engineering, Kogakuin University, 2665-1 Nakano-machi, Hachioji, Tokyo 192-0015, Japan

---

* Corresponding author. Tel: +81-42-628-4568; fax: +81-42-628-4568.

E-mail: shiro-seki@cc.kogakuin.ac.jp (S. Seki)

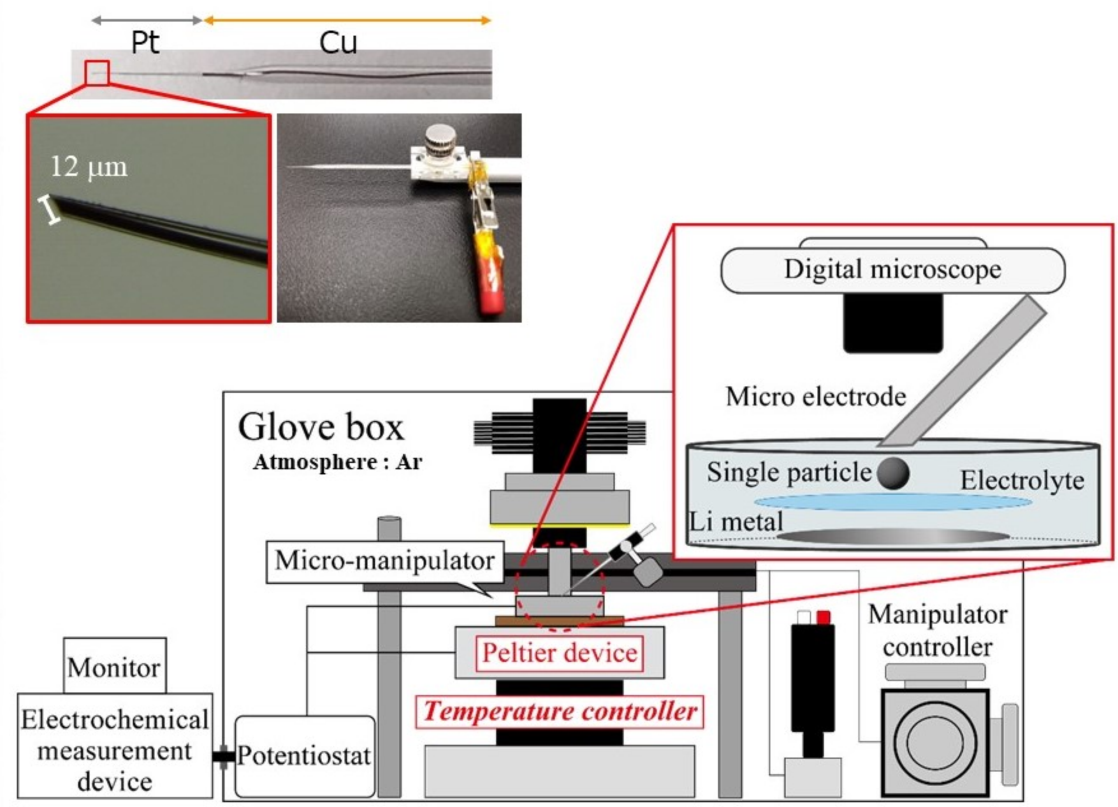

Figure S1. Schematic image of an SPEM cell used in this study.

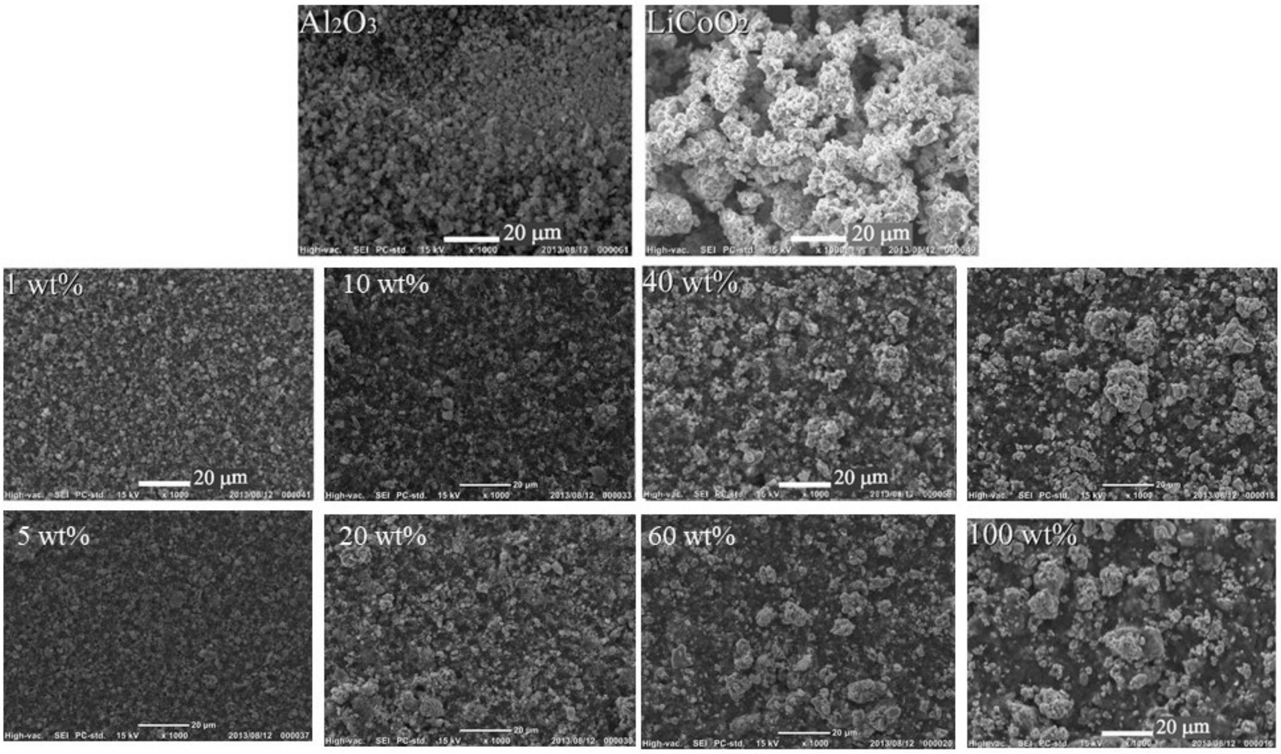

Figure S2. The SEM images of $LiCoO_2$, Al_2O_3 powder and surface of dilute-type applied positive electrode sheets.

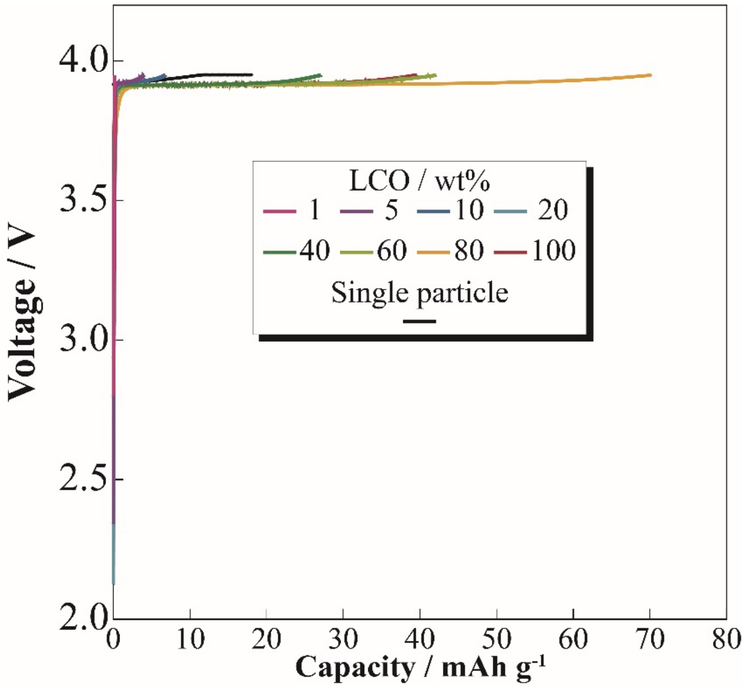

Figure S3. The charging curves of [Li|1.0 mol kg^{-1} LiFSA / EC| LCO-Al_2O_3] cell using dilute-type applied positive electrode sheet for 3.95 V. Capacities were also calculated based on the sum weight of LCO and Al_2O_3.
